# Resilience in the face of pandemic: exploring the influence of psychological flexibility on turnover intentions and burnout among critical care nurses in COVID-19 hospitals

**DOI:** 10.1186/s12912-024-02039-z

**Published:** 2024-07-10

**Authors:** Ayman Mohamed El-Ashry, Mohamed Mahmoud Seweid, Mohamed Adel Ghoneam, Sally Mohammed Farghaly Abdelaliem, Elsayed Mahmoud Sabek

**Affiliations:** 1 Psychiatric and Mental Health Nursing, Faculty of Nursing, Alexandria University, Alexandria, Egypt; 2 Faculty of Nursing, Beni-Suef University, Beni-Suef, Egypt; 3 Nursing Administration Department, Faculty of Nursing, Alexandria University, Alexandria, Egypt; 4 Faculty of Nursing, Beni Suef National University, Beni-Suef, Egypt

**Keywords:** Psychological flexibility, Burnout, Turnover intention, Critical care nurses, COVID-19

## Abstract

**Aim:**

Assess the levels of psychological flexibility, burnout, and turnover intention among critical care nurses and assess the impact of psychological flexibility on burnout, and turnover intention among critical care nurses.

**Background:**

Burnout and turnover intentions among critical care nurses are rapidly increasing because of the challenges of COVID-19. There is a need for evidence-based interventions like psychological flexibility to be addressed in research to overcome those challenges.

**Methods:**

A descriptive correlational research. A convenient sample of 200 critical care nurses working in COVID-19 hospitals from two governorates in Egypt. The sociodemographic and clinical data sheet, the work-related acceptance and action questionnaire, the Copenhagen burnout inventory, and the adopted version of the staff nurses’ intention to leave the nursing profession questionnaire were used.

**Results:**

The majority of critical care nurses reported a moderate level of psychological flexibility (75.5%, Mean = 31.23), a moderate level of burnout (65.5%, Mean = 59.61), and low to moderate levels of intention to leave (73%, Mean = 5.95). Psychological flexibility has a statistically significant negative correlation with burnout (PC = -0.304, Sig = 0.000) and the intention to leave (PC = -0.258, Sig = 0.000). In addition, psychological flexibility has a predictable effect on decreasing burnout (R^2^ = 0.232) and intention to leave (R^2^ = 0.127) among critical care nurses.

**Conclusion:**

critical care nurses in COVID-19 hospitals reported varied levels of burnout and an intention to leave that must be considered. The effect of psychological flexibility on burnout and intention to turnover highlighted the importance of improving it among critical care nurses by applying acceptance and commitment therapy as a management intervention.

## Introduction

A COVID-19 epidemic first surfaced in Wuhan, Hubei Province, China in December of 2019 [1]. In a matter of weeks, the virus outbreak started to spread horrifyingly throughout Wuhan and all of China [2]. When the number of afflicted countries reached 114, the World Health Organization proclaimed COVID-19 to be a global pandemic due to the absence of constraints and the infection’s quick spread [3]. At the beginning of April 2020, the Ministry of Health in Egypt reported that there were over 800 confirmed cases of COVID-19 and over 50 deaths, with a potential for the number to rise quickly [4]. The number of reported cases skyrocketed in just one month, reaching 6465 infected patients and almost 430 deaths on May 3, 2020. The population as a whole may experience dread and worry due to the astoundingly large increase in numbers in such a short amount of time [5].

Numerous investigations into the psychological effects of COVID-19 outbreaks on individuals have revealed a vast array of psychiatric issues [6]. Commonly expressed emotions included powerlessness, burnout, increased self-blame, and concern about contracting an infection or being sick. In addition, there are high rates of sickness and mortality, imprecise resources, stigma, gossip, and prejudice [7]. Furthermore, further research has to be done on how COVID-19 affects medical professionals that work in hospital settings, such as nurses, doctors, and other health workers.

A serious and fatal condition, nursing burnout impacts patients, organizations, and individual nurses. Up to 50% of nurses may experience burnout, which can have serious personal consequences, interfere with work, and harm patients [8]. People who are burned out may become weary while trying their hardest to assist patients who have little hope of recovery. Those who are burned out as clinicians believe they are not performing their jobs to the best of their ability; they also lack motivation and have low self-esteem in their profession [9]. When a patient is near death, nurses may bear a great emotional weight that affects their feelings [9]. Research on burnout has identified six occupational risk factors: a high workload, a lack of autonomy and independence, a lack of rewards and recognition, a lack of support and interpersonal conflict, a sense of justice, and the importance or values of the work [10]. The intention to leave nursing may increase because of all the previously listed circumstances.

Most organizations have a high turnover intention rate, which is linked to unfavorable employee outcomes [11]. Any professional move is referred to as turnover (Roche et al., 2015). They divided migration into two categories: internal, where nurses migrate between nursing units within the same hospital, and external, where they leave the profession or organization completely [12]. The quality of care is impacted by the high cost of nurse turnover. Developing tactics to combat turnover may be aided by an understanding of the relationship between organizational factors and the intention to leave [13]. In addition to aggravating the shortage of nurses by increasing work-related health issues and poor treatment quality, nurse turnover raises costs since new hires are less productive and temporary replacements must be hired, which adds to the financial burden [14]. Therefore, to improve staff retention and lessen the nursing staff shortage, nurse managers need have a thorough awareness of the reasons behind nurses’ intents to leave their jobs or profession. Job demands, work environment, social support, organizational culture, demographic characteristics, job satisfaction, and burnout were the factors that influenced nurses’ intention to leave [15, 16].

Thus, identifying the psychological mechanisms supporting the maintenance of psychological health and well-being in such atypical circumstances is critical. By having a greater understanding of these processes, we can help critical care nurses (CCNs) manage the current epidemic and plan appropriate interventions for the next months to prevent early psychological disorders from developing into serious, long-term mental health issues. A transdiagnostic, interdependent term, psychological flexibility encompasses a wide range of intrapersonal and interpersonal abilities. Since it resembles the idea of resiliency somewhat, it is seen as one of the primary components of mental health [17]. Numerous studies have shown that persons with high psychological flexibility will fare better in a variety of mental health situations [18–20]. According to Gloster et al.‘s (2017) research, psychological flexibility is crucial in modulating the link between everyday stress and outcomes related to physical and mental health and well-being in the general public [18].

According to some theories, the concept of psychological flexibility in Acceptance and Commitment Therapy (ACT) is founded on functional contextualism, which focuses on people’s capacity for resilience and effective functioning in the face of distress and is the primary goal of ACT [21]. In healthcare settings, a novel evaluation instrument has been employed to characterize and validate psychological flexibility associated to employment, allowing researchers to create stress-reduction strategies. Higher psychological flexibility was linked to reduced levels of psychological discomfort, such as stress, general psychological health, neuroticism, and emotional tiredness, according to research that employed this assessment instrument [22, 23].

Numerous studies examine the connection between burnout and the intention to leave in various healthcare environments [11, 14, 16]. Despite the global impact of COVID-19, a noticeable paucity of research has been conducted, especially in developing nations that need more funds for such studies. With a limited number of nurses available in these nations, assessing burnout, intention to leave, and psychological flexibility associated with employment among COVID-19 healthcare personnel is crucial. The present study aims to investigate the effectiveness of a novel intervention that utilized psychological flexibility, as demonstrated by acceptance and commitment therapy, to develop a dependable solution. Specifically, this study examines the levels of psychological flexibility, burnout, and turnover intention among CCNs working in COVID-19 institutions and the interrelationship between these variables.

## Design

Descriptive correlation was used in the study design.

### Setting

The study’s five critical care units were located in five governmental hospitals in Egypt: three in the Alexandria Governorate, Fever Hospital, ABOU-KIR General Hospital, and Kom El-Shokafa Chest Hospital; two in the Beni-Suef Governorate, Beni-Suef General Hospital, and Chest Hospital. COVID-19 patients are treated in these hospitals. All personal protection equipment (PPE), cardiac monitors, defibrillators, cardioverters, ventilators, and crash carts are provided in these hospitals’ intensive care units. The research was carried out in 2021 between January and March.

### Sample size calculation

The sample size calculation was performed using Epi-info 7 software, a widely accepted tool for epidemiological research. The parameters used were carefully chosen to ensure statistical validity: The anticipated prevalence or occurrence of the phenomenon under investigation was 50%. This was chosen as a conservative estimate to ensure that the sample size would be adequate regardless of the true frequency. The level of confidence desired in the study results is 95%. This indicates a 95% probability that the true population parameter lies within the calculated confidence interval. The maximum permissible difference between the sample estimate and the true population parameters is 5%. This ensures that the sample estimate remains sufficiently close to the true value for practical purposes. The total number of CCNs available for selection is 410 (190 at Beni-suef hospital and 220 at Alexandria hospital). This parameter provides context for the sample size calculation and ensures that the sample appropriately represents the population. The software determined that a minimum sample size of 198 was necessary to meet the specified criteria. However, a slightly larger convenient sample of 200 critical care nurses was ultimately included in the study to enhance statistical power and account for potential dropouts or non-responses [24].

In this study, convenient assignment was used as 200 CCNs were assigned to the two groups (Beni-Suef and Alexandrian COVID-19 hospitals) based on convenience factors, which could include factors like the nurses’ availability and proximity to the hospitals. One group consisted of 108 nurses who were assigned conveniently to the Beni-Suef hospitals. The other group consisted of 102 nurses who were assigned conveniently to the Alexandrian hospitals. It’s also mentioned that there were zero dropouts. This means that all the nurses who were assigned in both hospitals respond to the study tools, and none of them left or were excluded for any reason.

### Inclusion criteria

- Critical care nurses who are professionally qualified and actively engaged in providing care to critically ill patients in a hospital setting.
- Critical care nurses who have previously been involved in the direct care and treatment of patients diagnosed with severe cases of COVID-19.
- Critical care nurses who voluntarily agree to take part in the research study after being provided with information about its purpose, procedures, potential risks, and benefits.

### Exclusion criteria

- Critical care nurses who are not directly involved in providing bedside care to patients. This criterion helps to ensure that the participants have similar roles and responsibilities, which enhances the internal validity of the study.
- Critical care nurses who do not provide their voluntary assent to participate in the study.

### Tools of the study

An electronic questionnaire was used in this study. There were three sections to the survey. Sociodemographic and occupational characteristics, including age, gender, educational attainment, clinical experiences, workplace, and training or seminars addressing the virus, were covered in the first section. The second section included three tools.

#### Tool I: the work-related acceptance and action questionnaire (WAAQ)

The Work-Related Acceptance and Action Questionnaire (WAAQ) was developed by Bond et al. (2013) to evaluate psychological adaptability in the workplace [25]. It consists of seven items rated on seven points Likert statements, ranging from 1 (never true) to 7 (always true), such as “I can work effectively, even when I doubt myself” and “I can admit to my mistakes at work and still be successful.” The objects demonstrate people’s capacity for purposeful action even in the face of difficult internal circumstances. Higher WAAQ scores correspond to higher levels of psychological flexibility. The range of the scale is 7 to 49. Strong internal consistency was demonstrated when the WAAQ was used in a sample of medical professionals in Arabic study by El-Ashry et al., (2023) with Cronbach’s alpha of 0.84 [26].

#### Tool II: Copenhagen burnout inventory (CBI)

It was developed by Kristensen et al. (2005) and consisted of a 19-item questionnaire with three sub-dimensions: personal burnout (6 items), work-related burnout (7 items), and client-related burnout (6 items) [27]. The three portions of the questionnaire were designed to be applied in different situations. The purpose of the personal burnout questions was to make them universally understandable. It is implied by the questions regarding workplace burnout that the respondent has some experience working for pay. Finally, in the burnout questions related to clients, the term “client” (or a similar term when applicable, such as patient, student, inmate, etc.) emerges. The questions are easy to understand and answer, and the scales have a high face validity. There are five points on the Likert scale: 0% for never, very rarely, or to a very low degree; 25% for seldom, or to a low degree; 50% for occasionally, or somewhat; 75% for frequently, or to a moderate degree; and 100% for permanently, or to a very high degree. The possible score range for all scales is 0–100. With a Cronbach’s alpha score of 0.91, CBI is statistically significant [28].

#### Tool III: nurses’ intention to leave the nursing profession questionnaire

It was developed and validated by Peterson, (2009), then adapted and validated into Arabic by Ahmed et al., (2017) [29, 30]. It was consisted of six elements that were divided into two categories: one dealt with leaving the hospital setting and the other with leaving the medical field. Each segment had three items. The responses were rated on a three-point Likert scale (yes = 0, uncertain = 1, and no = 2). Based on cut points that indicate the degree of staff nurses’ intention to leave, the total scores were divided into three categories: low intention to leave (< 4), moderate intention to leave (< 8), and high intention to leave (< 12). The “staff nurses’ intention to leave the nursing profession questionnaire” has an acceptable reliability score of 0.813 [31].

## Methods

### Validity and reliability

A group of seven experts from Alexandria University evaluated the face and content validity of the instruments following their translation from English to Arabic using a forward-backward translation method. They also scrutinized the accuracy, clarity, and suitability of the Arabic translations of the research tools. Adjustments were implemented to ensure the tools reached their definitive valid versions. The Cronbach’s Alpha test was used to evaluate the internal consistency of the study instruments. The Staff Nurses’ Intention to Leave Nursing Profession Questionnaire, the CBI, and the WAAQ all showed excellent reliability, scoring 0.91, 0.82, and 0.79, respectively.

### Pilot study

A small-scale test was conducted with 20 CCNs, representing 10% of the study population. The primary goal of this test was to assess the usefulness and clarity of the study tools and identify any potential issues that may arise during data collection. In addition, the pilot aimed to estimate the time required to complete the tools and ensure the validity and reliability of the study instruments. It’s worth noting that the participants in the pilot were not part of the primary study sample. The pilot study results showed that the tools were clear and easy to understand, and no changes were required.

### Data collection

Once the nurses permitted their cell phone numbers to be used, they were invited to participate in the study through social media platforms like WhatsApp and Telegram. Before the online questionnaire link was shared, all nurses were informed about the study’s purpose and were asked to participate voluntarily. The online questionnaire was created for those with Microsoft accounts using Google Forms and Microsoft Forms. This questionnaire was shared as an online link, and the nurses were asked to complete it. Each nurse could only respond once to the online questionnaire. Electronic informed consent was obtained from each nurse on the first page of the questionnaire form. The purpose of the study was explained to them, and they were reassured about the anonymity and confidentiality of their responses. The data collection process spanned three months, from the beginning of July 2022 to the end of September 2022.

### Statistical analysis

After being gathered, the data were coded, modified, and then imported into IBM SPSS version 28, a statistical application. Cronbach’s alpha was employed to evaluate the reliability of the instruments. Frequency tables and cross-tabulation were used to illustrate the data.

A. The mean, standard deviation, minimum, and maximum of numerical data were all included in the descriptive statistical analysis. However, in categorical data, the percent was used to characterize the frequency of each group.
B. Inferential statistical analysis: The statistical link or connection between the three variables is assessed using the Pearson correlation test. The association between psychological flexibility at work, burnout, and intention to leave was examined using the Pearson correlation. There are three possible interpretations for the correlation coefficient: weak, moderate, and strong. A weak correlation, near 0, suggests minimal or no relationship between variables (e.g., below 0.30). A moderate correlation, around 0.50, indicates a moderate relationship. A strong correlation, nearing − 1 or 1, signifies a strong relationship, with high positive values indicating a direct relationship and low negative values indicating an inverse relationship [32]. *P*-values 0.05 was used to define statistical significance. In linear regression, the relationship between two variables is attempted to be modeled by fitting a linear equation to the observed data.

### Ethical consideration

The medical and nursing administrations of the Beni-Suef and Alexandria University hospitals gave their written approval for the study. The study’s settings were approved by Beni-Suef University’s Faculty of Medicine’s Research Ethical Committee (REC) in order to collect the required data. The participating nurses received a detailed explanation of the study’s objectives via email, along with assurances that the data would only be used for research. They were aware that there would be no consequences if they chose not to participate in the study or left before submitting the necessary paperwork. CCNs who volunteered to participate in the study gave electronic informed consent. We respected and considered everyone’s right to privacy. During the study, data privacy was protected as all responses was coded and protected by one of the researchers.

## Results

Table 1 shows the distribution of the studied subjects according to their socio-demographic and clinical data characteristics. The majority (82%) of the studied nurses was in the age group of less than 30 and was female (68.5%). In addition, half of the nurses who participated in the study were married (50%), and about 47% were single. According to the education received, more than half of the studied nurses get diplomas in nursing sciences (55%), and 41.5% get only a bachelor’s degree. Concerning work shifts, more than three-quarters of the nurses were working the day shift in the COVID-19 hospital (75.5%). According to years of experience, more than half of the studied nurses have less than five years of experience (59%). The table also showed that 33% had been infected with COVID-19 before, and 38% were suspected of being infected with COVID-19. In addition, the table shows that more than half of the family members (54% of the studied nurses) were infected with COVID-19.

**Table 1:** Distribution of the studied subjects according to their socio-demographic and clinical data

Socio-demographic and clinical data	Total (*N* = 200)	
	Frequency	%
**Age (years)**		
▪ < 30	164	82.0
▪ 30–40	33	16.5
▪ > 40	3	1.5
**Gender**		
▪ Female	137	68.5
▪ Male	63	31.5
**Marital status**		
▪ Married	100	50.0
▪ Single	94	47.0
▪ Divorced	3	1.5
▪ Widow	3	1.5
**Education**		
▪ Diploma	110	55.0
▪ Bachelor’s	83	41.5
▪ Graduate studies	7	3.5
**Shift**		
▪ Morning	18	9.0
▪ Evening	1	0.5
▪ Day shift	151	75.5
▪ Night	30	15.0
**Years of experience**		
▪ < 5 Years	118	59.0
▪ 5–10 Years	54	27.0
▪ 11–15 Years	10	5.0
▪ > 15 Years	18	9.0
**Previous COVID infection**		
▪ Confirmed	66	33.0
▪ Suspected	76	38.0
▪ No	58	29.0
**Family infection with COVID**		
▪ Yes	108	54.0
▪ No	92	46.0

The mean and standard deviation of nurses who worked at COVID-19 hospitals’ work-related psychological flexibility are shown in Table 2. The table indicates that with a mean score of 31.23 and a standard deviation of 5.367, the study’s nurses had moderate work-related psychological flexibility (75.5%).

**Table 2:** Distribution of mean and standard deviation the study subjects according to their work-related psychological flexibility

Psychological flexibility	Total (*N* = 200)			
	Frequency	%	Min.-Max.	Mean ± SD
▪ **Low**	13	6.5	13–42	31.23 ± 5.367
▪ **Moderate**	151	75.5		
▪ **High**	36	18.0		

Low PF (7–21) moderate PF (22–35) high PF (36–49)

Table 3 shows the study nurses’ mean and standard deviation distribution according to their feelings of burnout. The table shows that 63.5% of the studied nurses reported having a moderate level of personal burnout, with a mean score of 20.36. About 59% of the studied nurses also reported having a moderate level of work-related burnout, with a mean score of 22.8. Concerning client-related burnout, the table showed that 47.5% of the studied nurses reported having a moderate level, with a mean score of 16.39. The table also showed that about 65.5% of the studied nurses suffered from moderate burnout, with a mean score of 59.61.

**Table 3:** Distribution of mean and standard deviation of the study subjects according to their feeling of burnout

Burnout	Total (*N* = 200)			
	Frequency	%	Min.-Max.	Mean ± SD
**Personal burnout**				
▪ Low	11	5.5	11–30	20.36 **±** 4.097
▪ Moderate	127	63.5		
▪ High	62	31.0		
**Work-related burnout**				
▪ Low	22	11.0	9–35	22.87 ± 5.229
▪ Moderate	118	59.0		
▪ High	60	30.0		
**Client-related burnout**				
▪ Low	79	39.5	6–30	16.39 ± 5.403
▪ Moderate	95	47.5		
▪ High	26	13.0		
**Total burnout**				
▪ Low	23	11.5	29–95	59.61 ± 12.645
▪ Moderate	131	65.5		
▪ High	46	23.0		

Low burnout (19–44)  moderate burnout (45–69)  high burnout (70–95)

Table 4 shows the mean and standard deviation distribution among the study subjects according to their intention to leave nursing. The table shows that 38.5% of the studied nurses had a low intention to leave the hospital, and 36.5% had a moderate intention to leave the hospital, with a mean score of 3.13. Concerning the intention to leave the profession, the table shows that 45% of the studied nurses had a low intention to leave the profession, and 30.5% had a high intention to leave the profession, with a mean score of 2.82. The whole intention to leave shows that about 63.5% of critical care nurses who worked in COVID-19 hospitals had moderate to severe intention to leave.

**Table 4:** Distribution of mean and standard deviation of the study subjects according to their intention to leave the nursing profession

Intention to leave	Total (*N* = 200)			
	Frequency	%	Min.-Max.	Mean ± SD
**Intention to leave the hospital**				
▪ Low	77	38.5	0–6	3.13 ± 1.920
▪ Moderate	73	36.5		
▪ High	50	25.0		
**Intention to leave the profession**				
▪ Low	90	45.0	0–6	2.82 ± 2.234
▪ Moderate	49	24.5		
▪ High	61	30.5		
**Total intention to leave**				
▪ Low	73	36.5	0–12	5.95 ± 3.591
▪ Moderate	73	36.5		
▪ High	54	27.0		

Low intention to leave (< 4)  moderate intention to leave (4 < 8)  high intention to leave (8 − 12)

Table 5 shows the correlation matrix between the studies of CCNs in COVID-19 hospitals, work-related psychological flexibility, their feeling of burnout, and their intention to leave the nursing profession. The table shows the presence of a statistically moderate significant negative correlation between work-related psychological flexibility and the feeling of burnout (-0.304, 0.000). Also, a weak statistically significant negative correlation between work-related psychological flexibility and the intention to leave the nursing profession (-0.258, 0.000) was found. Finally, there was a moderate positive, statistically significant correlation between burnout and their intention to leave nursing (0.417, 0.000).

**Table 5:** Correlation between the study subjects’ work-related psychological flexibility, their feeling of burnout, and their intention to leave the nursing profession

Correlations (*N* = 200)				
		Work-related psychological flexibility	Feeling of burnout	Intention to leave
Work-related psychological flexibility	Pearson Correlation			
	Sig. (2-tailed)			
Feeling of burnout	Pearson Correlation	**-0.304** ^**^		
	Sig. (2-tailed)	**0.000**		
Intention to leave the nursing profession	Pearson Correlation	-0.258**	0.417**	
	Sig. (2-tailed)	**0.000**	**0.000**	

** Correlation is statistically significant at the 0.01 level (2-tailed)

Table 6 presents a linear regression analysis that explores the relationship between work-related psychological flexibility and feelings of burnout among the study subjects. The correlation coefficient is 0.434, indicating a moderately negative correlation between work-related psychological flexibility and feelings of burnout. This suggests that as work-related psychological flexibility increases, feelings of burnout decrease, and vice versa. The coefficient of determination (R^2^) is 0.232, which means that approximately 23.2% of the variation in feelings of burnout could be explained by work-related psychological flexibility. The model equation for this relationship was feeling of burnout = 81.966 − 0.716 x work-related psychological flexibility. This equation shows that for each unit increase in work-related psychological flexibility, burnout decreases by 0.716 units, assuming all other variables were held constant. These results were statistically significant (*p* ≤ 0.05), indicating a significant relationship between work-related psychological flexibility and feelings of burnout.

**Table 6:** Linear regression between the study subjects’ work-related psychological flexibility, and their feeling of burnout

Items	Feeling of burnout ^a^							
	*R*	*R* Square	F	Sig.	B	B1	T	Sig.
Work-related psychological flexibility	0.434^b^	0.232	20.145	0.000 *	81.966	-0.716	-4.488	0.000*

a. Dependent Variable: Feeling of burnout

b. Predictors: (Constant), Work-related psychological flexibility

F (ANOVA)  *Value of *p* ≤ 0.05 (significant)  Model Equation: Feeling of burnout = 81.966 − 0.716 X Work-related psychological flexibility

The linear regression analysis in Table 7 examines the relationship between work-related psychological flexibility and the intention to leave the nursing profession. The correlation coefficient 0.352 indicates a moderate positive relationship between these two variables. The R^2^ value of 0.127 suggests that work-related psychological flexibility could explain approximately 12.7% of the variation in the intention to leave nursing. The F statistic 14.159 is significant at *p* ≤ 0.05, indicating that the regression model predicts the dependent variable significantly. The regression coefficient (B1) of -0.173 suggests that for each unit increase in work-related psychological flexibility, the intention to leave the nursing profession decreases by 0.173 units, holding all other variables constant. The model equation, intention to leave the nursing profession = 11.346 − 0.173 x Work-related psychological flexibility, further illustrates this relationship. The constant (B) of 11.346 represents the predicted value of the intention to leave nursing when work-related psychological flexibility was zero.

**Table 7:** Linear regression between the study subjects’ work-related psychological flexibility, and their intention to leave the nursing profession

Items	Intention to leave the nursing profession ^a^							
	*R*	*R* Square	F	Sig.	B	B1	T	Sig.
Work-related psychological flexibility	0.352^b^	0.127	14.159	0.000 *	11.346	-0.173	-3.763	0.000*

a. Dependent Variable: Intention to leave the nursing profession

b. Predictors: (Constant), Work-related psychological flexibility

F (ANOVA)  *Value of *p* ≤ 0.05 (significant)  Model Equation: Intention to leave the nursing profession = 11.346 − 0.173 X Work-related psychological flexibility

## Discussion

This study examined the psychological flexibility, burnout, and intention to leave of CCNs who work in COVID-19 hospitals to determine whether these three characteristics were correlated.

The current study revealed that most CCNs employed in COVID-19 hospitals have moderate psychological flexibility related to their profession. It makes sense because the critical care unit puts more expectations and duties on the nurses, which leads to stress, burnout, and the intention to leave. To meet those obligations, nurses must learn self-management techniques. Acceptance is one of these tactics for situations that are unchangeable, particularly given the COVID-19 conditions in poor nations. This outcome was in line with research by Holmberg et al. (2020), which found that nurses in critical care units had a moderate level of work-related psychological flexibility [23].

It is necessary to address the moderate to severe degrees of burnout that the majority of the study’s participants reported having in relation to their personal, professional, and patient lives. Nurses who work in the front lines of treatment during the current COVID-19 health crisis are subjected to and confront severe and catastrophic mental and psychological crises, in addition to anxiety of getting the sickness and spreading it to their families [8]. The nurses will deal with continuous stress in addition to several fatal cases because of the ongoing COVID-19 alterations that will arise over time. Because they spend more time in proximity with sick patients than other professionals, nurses are more prone to burnout than other healthcare professionals, according to previous study [17]. The results of the current study were consistent with a study by de Cordova et al. that was carried out in 2022 among 3030 nurses in a New Jersey hospital and discovered that 64.3% of nurses encounter severe burnout. They also found that physical weariness, a shortage of nursing staff, and inadequate PPE were all contributing causes to burnout among nurses [33].

Numerous studies supported the current study’s conclusions regarding the degree of burnout experienced by nurses employed in COVID-19 facilities. Jose et al. (2020) found that there was moderate to high burnout in terms of depersonalization and emotional exhaustion among frontline nurses working in emergency rooms in India. However, burnout associated with personal accomplishment had less of an impact [34]. Frontline nurses were shown to have higher levels of burnout in another study conducted in Wuhan during the early stages of the COVID-19 pandemic. Specifically, 41.5, 27.5, and 38.3% of the participants reported emotional exhaustion, depersonalization, and personal achievement, respectively, as indicators of burnout [6].

Despite their best efforts, the studied nurses experienced moderate to severe burnout during the COVID-19 epidemic. Most expressed a low to moderate intention to leave their hospital jobs and careers. However, the nurses’ high psychological flexibility may have helped them cope with their burnout. Psychological flexibility refers to consistently engaging in activities that improve one’s health and quality of life, even amid pain or suffering, such as burnout [19]. It is important to note that the lower-than-average intention to leave among Egyptian nurses working in COVID-19 institutions is because they are compensated better and receive greater benefits compared to other specialty nurses. Additionally, these nurses may need help to search for a new workplace that can provide proper orientation and ensure compliance with new regulations [31]. The current study’s findings were in line with a study conducted in 2022 by de Cordova et al., which found that 36.5% of 3030 nurses stated they intended to leave the hospital within a year [31]. Prior to the pandemic, Ghandour et al.‘s study in Egypt revealed that over 50% of the nurses under investigation had a moderate inclination to leave [35].

Furthermore, a positive statistically significant correlation was found between burnout and the intention to leave, with the intention of research participants to leave nursing rising with larger levels of burnout. That was further supported by studies showing burnout is a strong predictor of the intention to leave [36, 37].

The results of the study showed that among CCNs working in COVID-19 facilities, psychological flexibility and burnout were correlated. Psychological flexibility was found to be a statistically significant predictor of decreased burnout among nurses, with a predicted proportion (R^2^ = 23.2%). This was consistent with a study done in 2021 by Sarabia-Cobo et al. on geriatric nurses, which discovered a connection between psychological flexibility and burnout [38]. Psychological flexibility is a protective quality that enhances compassion satisfaction and serves as an effective predictor of the development of burnout syndrome [39].

Additionally, the results showed that psychological flexibility has a statistically significant and predicted impact on the intention to leave (R^2^ = 12.7%) and is adversely connected with leaving. This was in line with research conducted by Holmberg et al. (2020), which examined the connection between medical staff members’ work engagement and psychological flexibility in intensive care units. They demonstrated how lower levels of work involvement were linked to higher levels of distress [23]. Additionally, there was a statistically significant indirect influence of psychological flexibility on the connection between distress and work engagement, and psychological flexibility was associated with more significant work engagement. Finally, a lower intention to leave and a higher level of work engagement were correlated with better psychological flexibility.

### Limitations

It is important to note the limitations of this study. Convenience sampling limits the generalizability of the study’s findings. Additionally, using self-reported metrics may lead to response bias. Secondly, a higher sample size was required for the investigation, which would have decreased the statistical power and the findings’ relevance. Our study’s strengths include the fact that, to our knowledge, this is the first study conducted in Egypt, mainly focused on the COVID-19 hospitals across two governorates from a nursing perspective.

## Conclusion

The study’s results are noteworthy and applicable to a variety of nursing scenarios, even though the sample was limited to Egyptian CCNs. In the present study, most CCNs employed in COVID-19 institutions demonstrate moderate to high levels of burnout, low to moderate levels of intention to leave, and moderate levels of psychological flexibility. Apart from its expected effect of lowering burnout and the intention to leave among CCNs employed at COVID-19 facilities, psychological flexibility was also adversely connected with burnout and the intention to leave. The influence that psychological flexibility had on the research variables demonstrated how important it is that CCNs use acceptance and commitment therapy as a management strategy to build this capacity.

### Implications in nursing practice

The study’s conclusions highlight important nursing practice considerations for assisting CCNs in navigating the pandemic’s obstacles. Stressing psychological flexibility in training programs can be crucial; these programs should emphasize acceptance and commitment therapy as well as stress-reduction techniques designed to meet the extreme demands placed on nurses in COVID-19 environments. Hospitals should place a high priority on identifying burnout early through regular assessments and preventative measures including workload distribution plans and counseling. It is critical to address elements that lead to burnout, such as a lack of employees and insufficient funding. To keep CCNs on staff, organizations should provide incentives, chances for professional growth, and work-life balance-enhancing programs.

## Data Availability

Data will be available on reasonable request from the corresponding author.

## Abbreviations

ACT: Acceptance and Commitment Therapy
CBI: Copenhagen Burnout Inventory
CCNs: Critical Care Nurses
PPE: Personal Protection Equipment
REC: Research Ethics Committee
WAAQ: Work Related Acceptance and Action Questionnaire
